# Developing and Testing the Reliability and Validity of the Brief Haze Weather Health Protection Behavior Assessment Scale-Adolescent Version (BHWHPBAS-AV)

**DOI:** 10.3389/fped.2020.498885

**Published:** 2020-09-22

**Authors:** Qingchun Zhao, Chun Yang, Shanshan Tang, Yuejia Zhao, Hongzhe Dou, Yanhong Chen, Yanrong Lu, Lingwei Tao

**Affiliations:** ^1^Outpatient Department, Operating Room, Blood Transfusion Department, Affiliated Hospital of Hebei University, Baoding, China; ^2^School of Public Health, Beijing Key Laboratory of Environmental Toxicology, Capital Medical University, Beijing, China; ^3^United Front Department, Hebei University, Baoding, China; ^4^School of Public Health, Peking University, Beijing, China

**Keywords:** adolescent, haze, health protection behavior, reliability, validity

## Abstract

**Objectives:** To develop a Brief Haze Weather Health Protection Behavior Assessment Scale-Adolescent Version (BHWHPBAS-AV).

**Methods:** Considering primary prevention, secondary prevention and tertiary prevention as a theoretical basis, researchers developed a Brief Haze Weather Health Protection Behavior Assessment Scale-Adolescent Version**-I(**BHWHPBAS-AV**-I)**. After performing 6 reviews by related experts, and after conducting six adolescent tests for BHWHPBAS-AV**-I**, researchers developed an updated BHWHPBAS-AV-II. Out of the 20 districts in Baoding, two districts were randomly selected; moreover, two middle schools from these two districts were also randomly selected. Considering one class as a unit, researchers subsequently randomly selected 22 classes by using stratified sampling. In the end, 1,025 valid questionnaires were used as part of the study. At which point, researchers investigated the validity and reliability of the scale and obtained the final scale (BHWHPBAS-AV).

**Results:** BHWHPBAS-AV Cronbach's α = 0.878, content validity = 0.948, and factor cumulative contribution rate = 54.058% using exploratory factor analysis. By confirmatory factor analysis, Chi square value (χ^2^) = 271.791, degrees of freedom (df) = 94, Chi square value/degrees of freedom (χ^2^/df) = 2.891, root-mean-square error of approximation (RMSEA) = 0.051, normed fit index (NFI) = 0.930, incremental fit index (IFI) = 0.953, goodness of fit index (GFI) = 0.955, Tueker-Lewis index (TLI) = 0.940, comparative fit index (CFI) = 0.953. BHWHPBAS-AV was composed of 16 items as well as 3 dimensions.

**Conclusions:** A BHWHPBAS-AV scale that has an acceptable reliability and validity can be applied to assess adolescent haze weather health protection behavior, and can also help school teachers, as well as medical staff working in community health care institutions, to perform targeted behavioral interventions and deliver health education programs to adolescents.

## Introduction

Air pollution has become a serious global problem, influencing the health level of millions of people around the world (1). In China, many serious public health problems are directly a result of air pollution. In 2012, the Chinese Government introduced its National Plan directed toward Air Pollution Control. The plan outlined stringent air pollution prevention goals and control measures. The Chinese Government promulgated the first National Action Plan focusing on Air Pollution Prevention and Control (2013–2017) in September 2013, which hoped to lead to a significant improvement in national air quality by 2017 (2). As an important manifestation of air pollution, haze weather may cause serious short-term harm, as well as have a long-term negative impact on human health. Subsequently, this may lead to further issues in public health, especially regarding the growth and development of children and adolescents (3–8). Owing to the fact that the adolescent period of life is a critical stage in growth and development, adolescent physiological functions remain immature and are thus susceptible to the effects of haze (1, 9–13). The particulate matter that contributes to haze weather can directly enter the respiratory system and subsequently adhere to the respiratory mucosa or deposit in the alveoli, which results in damage to the respiratory tract and alveolar epithelial cells. This may reduce the lung capacity of adolescents and cause asthma, rhinitis, bronchitis, pneumonia, and lung cancer (9, 11, 14). In addition, the pollutant in haze weather can weakens the immunity of adolescents, triggers allergic reactions, damages the circulatory and nervous system, and has the potential to give rise to metabolic diseases (15, 16). Compared with adults, adolescents have a lesser understanding of the harm that haze weather can cause; moreover, a lower proportion of the youth tend to adopt health protection behaviors in haze weather (16). Undoubtedly, it is critical for the safety of adolescents that we carry out social awareness campaigns to educate the public, especially adolescents, about haze weather, and how to protect oneself against its hazards (17). Implementing protective behaviors against haze weather can help prevent youngsters from suffering from haze weather related health conditions and can promote adolescent normal growth and development. Besides that, an increased social awareness will surely reduce the burden haze weather has on families, schools, the government, and society in general (18).

At present, the majority of research to do with haze weather is focused on the experimental research field, for example, its harmful effects on tissues and organs (9, 11, 12). However, the findings and implications from these experimental studies have not been widely related to the health protection behavior of adolescents (16). Therefore, it is important to develop a haze weather health protection behavior assessment scale for adolescents, in order to better encourage them to adopt protective health behaviors against haze weather. Deguen carried out a research in eight French cities with the aim of developing an air quality perception scale. Deguen's study indicated that the scale has good validity and reliability and can be used to understand the community residents' perceptions of air quality (19). Ye developed a questionnaire that explored the level of environmental health literacy, attitude, and habits of Beijing-Tianjin-Hebei region residents, surrounding the issue of haze weather (17). In Iran, Mirzaei-Alavijeh developed a self-care behavior related to air pollution protection questionnaire. The questionnaire is a promising instrument that assesses self-care behavior related to air pollution protection from the perspective of college students (20). However, currently, there is no haze weather health protection behavior assessment scale for adolescents. Therefore, our research aims to firstly develop a Brief Haze Weather Health Protection Behavior Assessment Scale-Adolescent Version (BHWHPBAS-AV), and secondly to test its validity and reliability. Wu pointed out that the conceptual framework of a scale should be based on previous theoretical literature or some exploratory empirical researches (21). Zhan pointed out that prevention strategies can be divided into three stages, which include primary prevention, secondary prevention and tertiary prevention (22). In our study, using these three stages of prevention as the theoretical basis, and by investigating related literature references (22–24), research team developed the Brief Haze Weather Health Protection Behavior Assessment Scale-Adolescent Version. BHWHPBAS-AV will help teachers in schools, as well as medical staff in health care institutions, to carry out health education and targeted behavioral interventions, for adolescents who are susceptible to the effects of haze.

## Methods

### Development of BHWHPBAS-AV-I

Considering primary, secondary and tertiary prevention as the theoretical foundation (22–24), in addition to investigating extensive literature references, researchers developed an initial scale (Brief Haze Weather Health Protection Behavior Assessment Scale-Adolescent Version-I, BHWHPBAS-AV-I). The initial scale included 20 items and 4 dimensions. Researchers named the four dimensions as follows: Dimension 1, the way to obtain relevant knowledge before haze weather; Dimension 2, the source of relevant knowledge before haze weather; Dimension 3, self-protection in haze weather; Dimension 4, self-adjustment after haze weather. These items of BHWHPBAS-AV-I were presented in way that was simple and easy to understand so that the participants were able to comprehend the meaning of each item with ease (25, 26).

### Development of BHWHPBAS-AV-II

Six experts (two clinical doctors, two clinical nurses and two health care experts) were invited to assess the scale content validity. The evaluation standard of content validity ranged from 1 (not related) to 3 (strongly related). Based on the expert reviews, four items from the BHWHPBAS-AV**-I** were removed, which resulted in an updated scale (BHWHPBAS-AV-II). As part of the BHWHPBAS-AV-II, 16 items and 4 dimensions were included. The names of each of the dimensions remained unchanged. BHWHPBAS-AV-II used the Likert 5-point method (1 = disagree; 2 = agree a small part; 3 = moderately agree; 4 = agree most; 5 = completely agree). The total score of the scale was determined by the sum of items' scores. A higher total score suggested better adolescent haze weather health protection behavior. Subsequently, the research team asked 6 adolescents to complete BHWHPBAS-AV-II so that researchers could test and possibly improve the wording and comprehension of statement expressions. Every item in the BHWHPBAS-AV-II was presented in plain and simple to understand language so that the adolescent participants could easily comprehend the meaning of the items (25, 26).

### Development of the Final BHWHPBAS-AV and Large Sample Test

**Figure 1:**
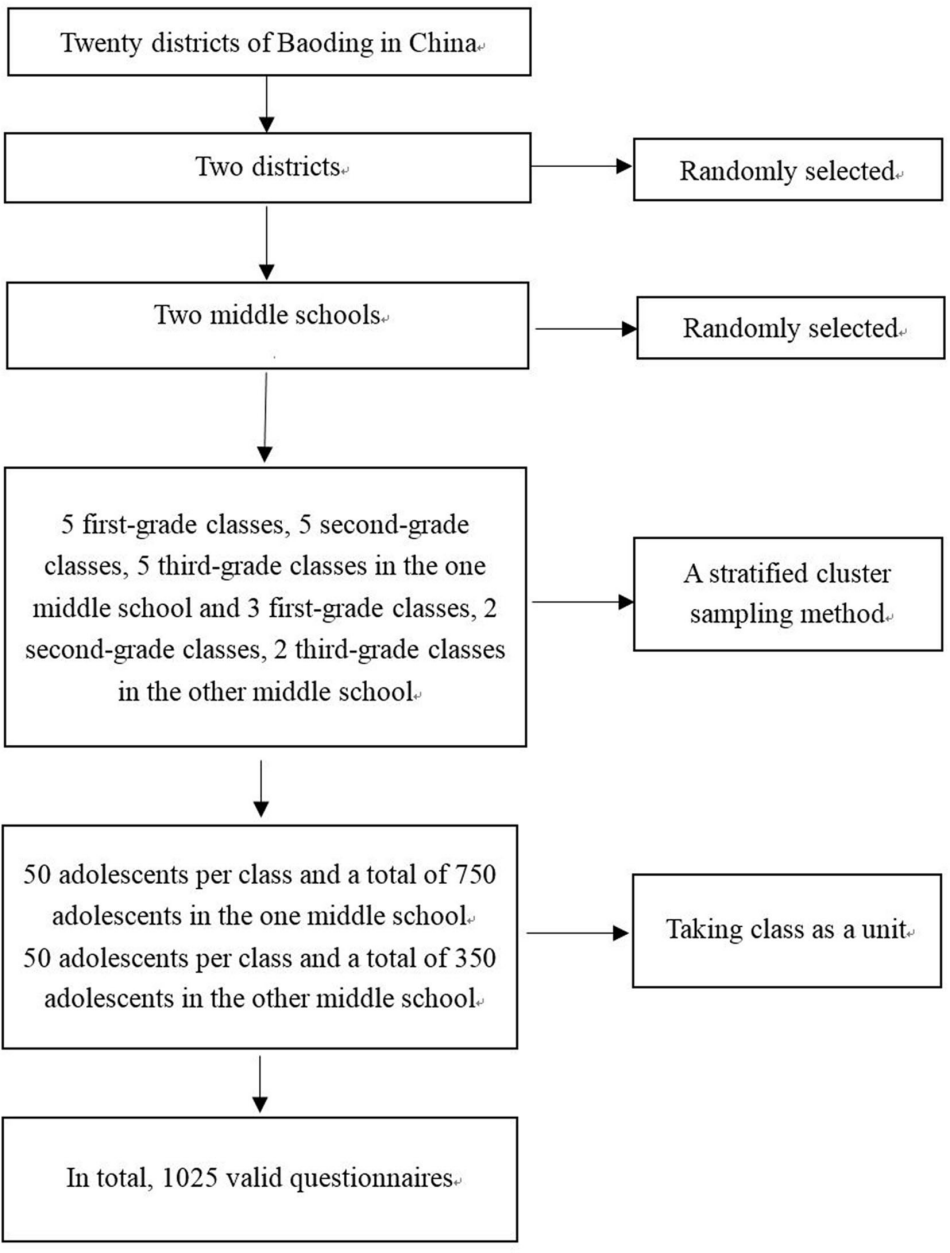
Study participant flow diagram.

From June 2015 to April 2016, in Baoding, China, the research team randomly selected two districts from the twenty districts of Baoding. Subsequently, researchers randomly selected two middle schools from these two districts. Considering a class as a unit, and by conducting stratified sampling, the research team randomly selected five first-grade classes, five second-grade classes, and five third-grade classes from the one middle school (a total of 750 adolescents and 50 adolescents per class). In addition, three first-grade classes, two second-grade classes, and two third-grade classes were selected from the second middle school (a total of 350 adolescents and 50 adolescents per class). In total, this study involved 1100 adolescents. Inclusion criteria: (1) Adolescents have a satisfactory capacity to comprehend, no intellectual disabilities and no reading disabilities; (2) Participants volunteered for the study; (3) Participants do not suffer from any organic brain disease, mental condition or other serious health problems. Sample size: Both exploratory factor analysis and confirmatory factor analysis were appropriately used when the hypothesized measurement model was evaluated. The sample size should be at least 10-15 individuals per item for the factor analysis (27). If the sample size was more than 20 individuals per item for the factor analysis, the results of factor analysis would be more stable and more reliable (28). Since this scale contained 16 items, the sample size of the large sample test should be more than 320. However, to produce more stable reliable results, after comprehensively considering the feasibility of the study, we appropriately increased the number of samples. Ultimately, the research team distributed 1,100 questionnaires. Twenty-one of the participants did not complete demographic characteristic questionnaires or scales, and 54 participants did not complete the scale. Thus, these 75 participants were excluded. Ultimately, 1,025 valid questionnaires were returned. Valid recovery rate = 1,025 valid questionnaires ÷1,100 total questionnaires = 93.18%. Therefore, the valid recovery rate was 93.18%. After analyzing data from 1,025 valid questionnaires, we tested the validity and reliability of the scale and developed the final version of the scale (BHWHPBAS-AV). The study participant flow diagram is shown in Figure 1.

### Ethical Consideration and Survey Method

The study was approved by the Health and Family Planning Commission of Hebei Province (NO.20150072). The study was also approved by the Medical Ethics Committee of Hebei University. This study was carried out according to the standards set by the Declaration of Helsinki. Researchers explained the purpose of research to the middle school teaching management departments, parents/guardians of the minors, as well as the adolescents themselves. After receiving consent from the school coordinators, parents/guardians of the minors and the adolescents themselves, the research team demonstrated to the participants how to answer the questionnaires. The questionnaires used standardized language and unified instruction.

### Statistical Analysis

In order to complete a data consistency check, the research team used the Epidata 3.1 software to input the data into the computer twice. The data were analyzed using the AMOS 17.0 software and SPSS 18.0 software. The research team used descriptive statistics (frequency/percentages or medians/interquartile ranges) to explore adolescent demographic characteristics. The following list represents the methods we used for measuring the validity and reliability of scale: The exploratory factor analysis (EFA) and the confirmatory factor analysis (CFA) were used to assess construct validity; Content validity index (CVI) was applied to assess content validity of the scale; Mean inter-item correlation coefficient (MIIC) and Cronbach's α coefficient were used to evaluate the reliability of the scale. This study set the level of significance at *p* < 0.05. The following criteria were used for the retention of factors: Eigenvalues > 1; EFA scree plot; Items equal to, or >2 being retained; and The factor loadings > 0.500 (29). The research team considered the model fit of scale structure as acceptable if root-mean-square error of approximation (RMSEA) <0.08, Chi square value/degrees of freedom (χ^2^ /df) <3, normed fit index (NFI) >0.90, goodness of fit index (GFI) >0.90, Tueker-Lewis index (TLI) >0.90, incremental fit index (IFI) >0.90 and comparative fit index (CFI) >0.90 (26, 29, 30). The factor loading cut-off point for CFA was 0.50 (28).

## Results

### Characteristics Pertaining to the Large Sample of Adolescents

A total of 1,100 questionnaires were distributed to the study participants, and 1,025 satisfactory questionnaires were returned. The valid recovery rate of the questionnaires was 93.18%. Subjects included 515 men (50.2%) and 510 women (49.8%) from urban (66%) and rural areas (34%). The age of adolescents was 14 (13, 16) years old, medians and interquartile ranges (IQR). Participant demographics included Han race (96.6%) and minority race (3.4%). Adolescents were categorized into three groups according to monthly expenses: <300 yuan, 300–599 yuan and ≥600 yuan, accounting for 33.4, 52.4, and 14.2%, respectively. Adolescents were also classified into three groups according to the method of medical insurance they used, including urban medical insurance, new rural cooperative medical system and self-paying, accounting for 58.0% 29.9 and 12.2% respectively. The data characteristics of the large sample of adolescents are shown in Table 1 in detail.

**Table 1:** The characteristics data of the large sample adolescents.

**Characteristics**	**Frequency/Medians**	**Percentage (%)/Interquartile ranges, (IQR)**
**Gender**		
Male	515	50.2
Female	510	49.8
Age	14	13, 16
**Race**		
Han	990	96.6
Minority	35	3.4
**Monthly expenses (yuan)**		
<300	342	33.4
300~	537	52.4
600~	146	14.2
**Do you have a religious faith**		
No	952	92.9
Yes	73	7.1
**Place of residence**		
Urban area	677	66.0
Rural area	348	34.0
**Way of medical insurance**		
Urban medical insurance	594	58.0
New rural cooperative medical system	306	29.9
Self-paying	125	12.2
**Do you live with your family**		
Yes	958	93.5
No	67	6.5

*The characteristics data of the large sample were presented as frequency (percentage) or medians (interquartile ranges, IQR)*.

### Analysis of Reliability and Validity

#### Construct Validity

##### Exploratory factor analysis (EFA)

**Figure 2:**
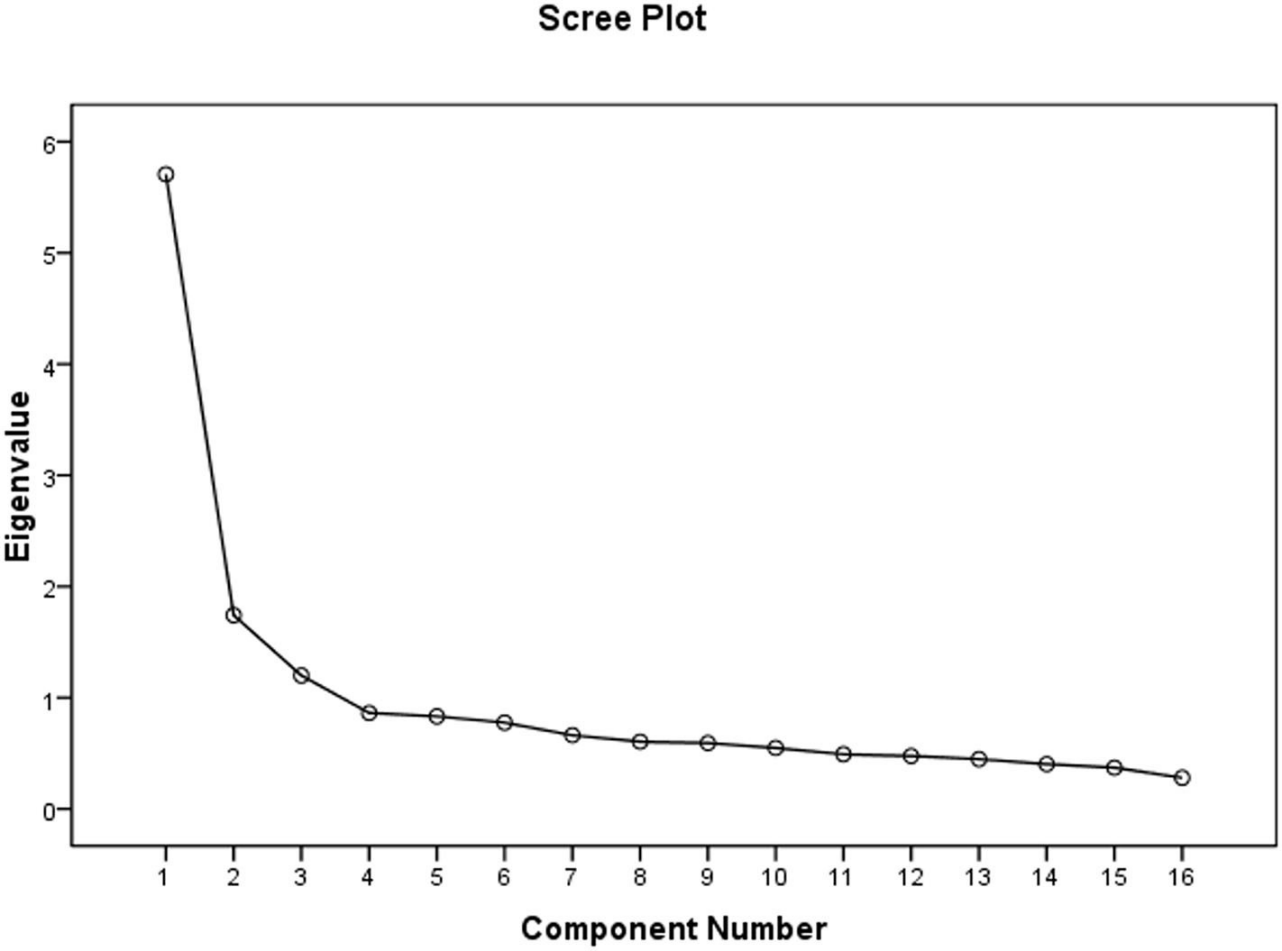
Scree plot of exploratory factor analysis.

The research team analyzed the BHWHPBAS-AV-II of 1,025 subjects. Principal component analysis (PCA) and maximum variance orthogonal rotation were applied to conduct EFA of BHWHPBAS-AV-II. The results revealed that the Bartlett sphericity test value was 5668.801 (df = 120, *p* < 0.001) and the Kaiser-Meyer-Olkin value (KMO) was 0.895. The results indicated that the scale data were suitable for factor analysis. Factor extraction was conducted under a condition of undefined factor number. Three factors (Eigenvalue >1) were extracted and the cumulative variance contribution rate (%) was 54.058%. The scree plot of BHWHPBAS-AV-IIfactor analysis indicated an inflection point between the 3rd factor and the 4th factor. The scree plot of exploratory factor analysis also revealed that the 3-factor structure was suitable (Figure 2). After performing the aforementioned analyses, the final version of the scale (BHWHPBAS-AV) included three factors and 16 items. Researchers renamed the final three factors: Factor 1, obtain relevant knowledge before haze weather (7 items); Factor 2, self-protection in haze weather (four items); Factor 3, self-adjustment after haze weather (five items) (Table 2). The detailed content of Brief Haze Weather Health Protection Behavior Assessment Scale-Adolescent Version (BHWHPBAS-AV) is shown in Table 3.

**Table 2:** Rotated component matrix, eigenvalue and cumulative variance contribution rate.

	**Items**	**Factor 1**	**Factor 3**	**Factor 2**
	Q1	0.624	_	_
	Q2	0.641	_	_
	Q3	0.685	_	_
	Q4	0.714	_	_
	Q5	0.744	_	_
	Q6	0.742	_	_
	Q7	0.583	_	_
	Q8	_	_	0.795
	Q9	_	_	0.817
	Q10	_	_	0.737
	Q11	_	_	0.554
	Q12	_	0.702	_
	Q13	_	0.560	_
	Q14	_	0.589	_
	Q15	_	0.691	_
	Q16	_	0.714	_
Eigenvalue		5.707	1.742	1.200
Variance contribution rate (%)		35.670	10.890	7.498
Cumulative variance contribution rate (%)		35.670	46.560	54.058
Factor naming		Obtain relevant knowledge before haze weather	Self-adjustment after haze weather	Self-protection in haze weather

*Factor 1, obtain relevant knowledge before haze weather; Factor 2, self-protection in haze weather; Factor 3, self-adjustment after haze weather. This symbol “_” indicated that values were <0.500. Suppress absolute values <0.500*.

**Table 3:** The content of Brief Haze Weather Health Protection Behavior Assessment Scale-Adolescent Version (BHWHPBAS-AV).

**Dimensions**	**Items**	**Completely agree 5**	**Agree most 4**	**Moderately agree 3**	**Agree a small part 2**	**Disagree 1**
Obtain relevant knowledge before haze weather	Q1. I often learn the health protection knowledge of haze weather through television media.	5	4	3	2	1
	Q2. I often learn the health protection knowledge of haze weather through Internet media.	5	4	3	2	1
	Q3. I often learn the health protection knowledge of haze weather through related books.	5	4	3	2	1
	Q4. I often learn the health protection knowledge of haze weather through school related publicity activities.	5	4	3	2	1
	Q5. I often learn the health protection knowledge of haze weather through my teacher.	5	4	3	2	1
	Q6. I often learn the health protection knowledge of haze weather through my classmates.	5	4	3	2	1
	Q7. I often learn the health protection knowledge of haze weather through my parents.	5	4	3	2	1
Self-protection in haze weather	Q8. When the haze weather happens, I clean my nasal cavity in time.	5	4	3	2	1
	Q9. When the haze weather happens, I clean my eyes in time.	5	4	3	2	1
	Q10. When the haze weather happens, I clean my face in time.	5	4	3	2	1
	Q11. When the haze weather happens, I wear a protective mask.	5	4	3	2	1
Self-adjustment after haze weather	Q12. After haze weather is over, I eat more fruits and more vegetables.	5	4	3	2	1
	Q13. After haze weather is over, I eat less spicy and irritating food.	5	4	3	2	1
	Q14. After haze weather is over, I increase my physical exercise time.	5	4	3	2	1
	Q15. After haze weather is over, I participate in some recreational activities to relax.	5	4	3	2	1
	Q16. After haze weather is over, I increase my sleep.	5	4	3	2	1

##### Confirmatory factor analysis (CFA)

**Figure 3:**
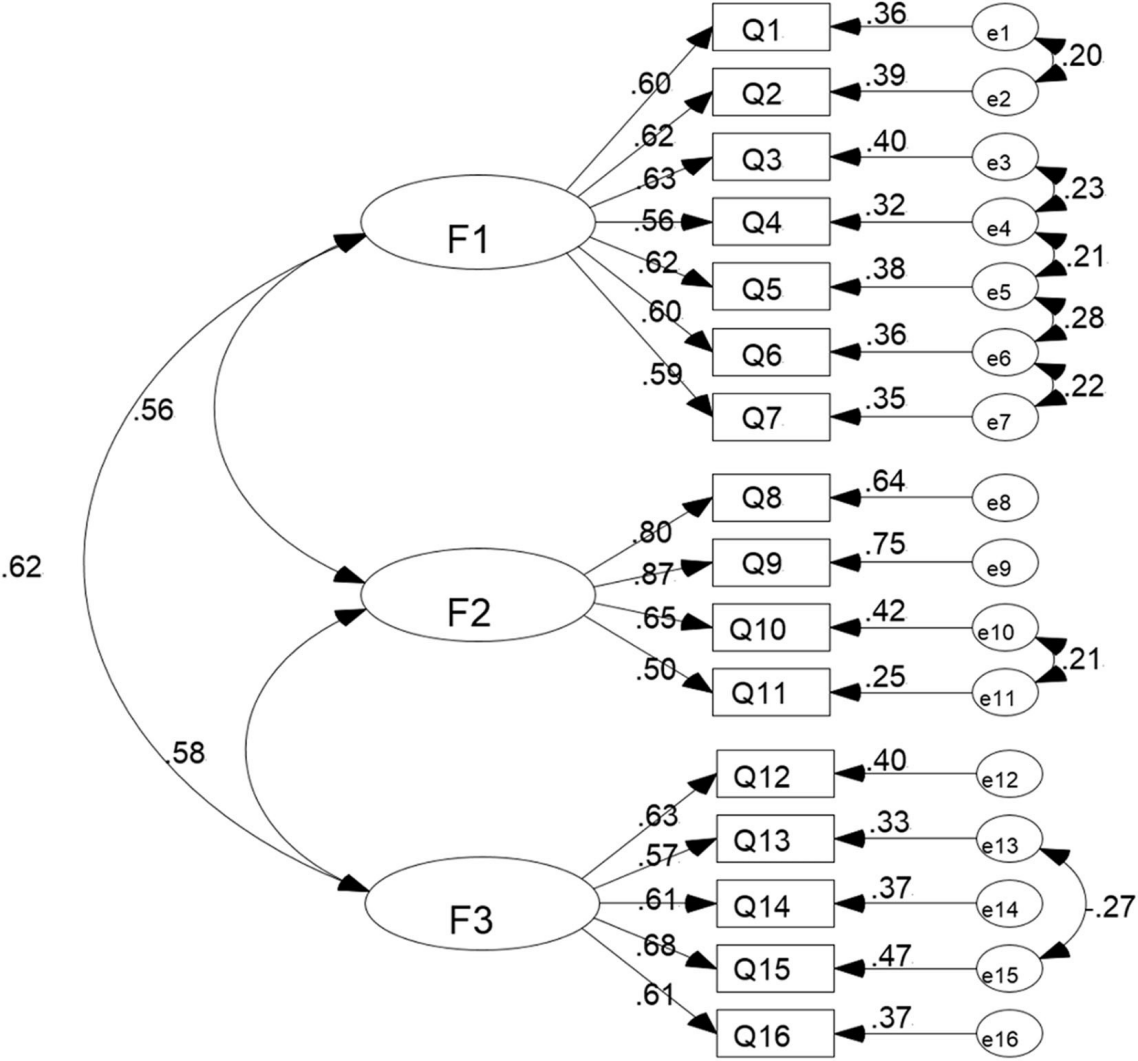
Standard path and parameter estimation of confirmatory factor analysis. F1, Factor 1, obtain relevant knowledge before haze weather; F2, Factor 2, self-protection in haze weather; F3, Factor 3, self-adjustment after haze weather.

In order to confirm the best dimensional structures of BHWHPBAS-AV, using AMOS17.0 software, the research team randomly selected 70% of samples (718 subjects) and applied the maximum likelihood method to conduct the CFA of 16-item, and 3-factor structures of BHWHPBAS-AV. The Chi square value (χ^2^) was 271.791, degrees of freedom (df) was 94, Chi square value/degree of freedom (χ^2^/df) was 2.891, root-mean-square error of approximation (RMSEA) was 0.051, goodness of fit index (GFI) was 0.955, normed fit index (NFI) was 0.930, incremental fit index (IFI) was 0.953, Tueker-Lewis index (TLI) was 0.940, comparative fit index (CFI) was 0.953 (Table 4). The standard path and parameter estimation of CFA is shown in Figure 3.

**Table 4:** The results of confirmatory factor analysis.

***χ^2^***	**df**	***χ^2^* /df**	**RMSEA**	**GFI**	**NFI**	**IFI**	**TLI**	**CFI**
271.791	94	2.891	0.051	0.955	0.930	0.953	0.940	0.953

*χ^2^, Chi square value; df, degrees of freedom; χ^2^/df, Chi square value/degrees of freedom; RMSEA, root-mean-square error of approximation; GFI, goodness of fit index; NFI, normed fit index; IFI, incremental fit index; TLI, Tueker-Lewis index; CFI, comparative fit index*.

#### Internal Correlation Test

Among the factors of BHWHPBAS-AV, the correlation coefficients ranged from 0.458 to 0.547 (*p* < 0.01). Correlation coefficients between all factors and the whole scale of BHWHPBAS-AV ranged from 0.798 to 0.812 (*p* < 0.01) (Table 5).

**Table 5:** Correlation coefficients among the factors of BHWHPBAS-AV and between the factors and the whole BHWHPBAS-AV.

**Factor**	**Factor 2**	**Factor 3**	**BHWHPBAS-AV**
Factor 1	0.458^**^	0.467^**^	0.812^**^
Factor 2	_	0.547^**^	0.798^**^
Factor 3	_	_	0.809^**^

*Factor 1, obtain relevant knowledge before haze weather; Factor 2, self-protection in haze weather; Factor 3, self-adjustment after haze weather. This symbol “_” indicated no such correlation coefficient*.

** *p < 0.01*.

#### Content Validity

According to the findings obtained from six expert reviews, the content validity index (CVI) of scale was 0.948. The CVI of every item ranged from 0.667 to 1.00. After improving the item statement expression and the item wording, six participants reported that they could clearly understand the meanings of all items without any difficulty.

#### Reliability

The BHWHPBAS-AV mean inter-item correlation coefficient value (MIIC) was 0.312. The MIIC values for all factors ranged from 0.368 to 0.498. The BHWHPBAS-AV Cronbach's α coefficient value was 0.878, and the Cronbach's α coefficients of all factors ranged from 0.744 to 0.837 (Table 6).

**Table 6:** The MIIC and the Cronbach's α coefficient of each factor and the total BHWHPBAS-AV.

**Factor**	**Number of Items**	**MIIC**	**Cronbach's α**
Factor 1	7	0.424	0.837
Factor 2	4	0.498	0.799
Factor 3	5	0.368	0.744
BHWHPBAS-AV	16	0.312	0.878

*Factor 1, obtain relevant knowledge before haze weather; Factor 2, self-protection in haze weather; Factor 3, self-adjustment after haze weather. MIIC, mean inter-item correlation coefficients*.

## Discussion

Nowadays, medical systems globally, are gradually changed from the old health care model, which regarded medical staff as leading decision makers in patient care, to a newer and more patient centralized health care model, which involves both medical staff and individuals in the decision making process of a patient's health management (31, 32). Therefore, by significantly improving adolescent health protection ability against haze weather, and by fully mobilizing adolescents' enthusiasm of health protection, the detrimental health impact caused by haze weather may be significantly reduced. The current guidelines, which China's medical system adopts, predominantly focus on the diagnosis, treatment, and care of specific diseases. However, it does not places enough importance on investing in health education of families, schools, and communities. Adolescent knowledge regarding the harmful impact of haze weather is inadequate, and their health protection behaviors pertaining to this issue need to be further improved. The BHWHPBAS-AV developed in our research enables teachers at schools, as well as medical staff in community health care institutions, to effectively and rapidly asses the degree of haze weather health protection behaviors that adolescents have. Teachers at school, as well as the medical staff in the community, will be able to carry out targeted behavioral interventions and provide adolescents with relevant and necessary health education. These measures will help adolescents develop good health protection habits against the harms that haze weather causes. This will reduce the incidence of related diseases. Finally, this will also reduce the relevant medical burden placed on families, schools, and government bodies (33).

In this study, when researchers needed to asses a hypothesized measurement model, both exploratory factor analysis and confirmatory factor analysis are appropriately applied (27). The sample size in the study should involve at least 10–15 participants per variable for the factor analysis of the scale (27). Our sample size was large enough for the analysis. In the exploratory factor analysis, in order to evaluate the suitability of factor analysis, the research team conducted the Bartlett's test of sphericity and calculated the KMO value. Bartlett's test of sphericity (5668.801, df = 120, *p* < 0.001) was significant, and the KMO value (0.895) in our research was >0.6 (34). The aforementioned findings revealed that the collected data were suitable for factor analysis. Exploratory factor analysis suggested that 16 items loaded substantially onto three conceptually clear factors. Dimension 1 (the way to obtain relevant knowledge before haze weather) and dimension 2 (the source of relevant knowledge before haze weather) in the BHWHPBAS-AV-II were merged into the dimension 1 (obtain relevant knowledge before haze weather) to create the final version of BHWHPBAS-AV. The reason for changing the two dimensions was because, essentially, dimension 1 (the way to obtain relevant knowledge before haze weather) and dimension 2 (the source of relevant knowledge before haze weather) both measure the status of obtaining relevant knowledge prior to the occurrence of haze weather. Therefore, finally, researchers merged dimension 1 (the way to obtain relevant knowledge before haze weather) and dimension 2 (the source of relevant knowledge before haze weather) into dimension 1 (obtain relevant knowledge before haze weather). This helped the BHWHPBAS-AV structure become clearer, more concise, and easier to use. For the confirmatory factor analysis, the model goodness of fit is assessed by RMSEA (<0.08 acceptable), IFI (>0.90 acceptable), GFI (>0.90 acceptable), NFI (>0.90 acceptable), CFI (>0.90 acceptable), and TLI (>0.90 acceptable) (26, 29, 30). All the results of confirmatory factor analysis in this research met the above criteria. The results of confirmatory factor analysis show that both the stability and the fit of the 3-factor model structure of BHWHPBAS-AV are good.

Typically, content validity indicates whether items of a scale are able to identify the topic and content that the researcher aims to measure. The CVI of an item represents the number of expert choices of 3 and 2 divided by the total number of experts. The total CVI value of a scale is the average value of all of items' CVI values (26). The total CVI value of scale in this study was 0.948. The CVI values of all items ranged from 0.667 to 1.00. This indicates that BHWHPBAS-AV is able to reflect the variables measured. Every item was able to measure the correct content, and the BHWHPBAS-AV has shown a satisfactory content validity. The internal correlation test results of BHWHPBAS-AV showed that there was a certain degree of correlation between these factors; furthermore, there were also some differences between these factors. Thus, these factors were able to reflect different aspects of adolescent haze weather health protection behaviors. Thus, the three factors were able to effectively and comprehensively assess the status of adolescent haze weather health protection behaviors.

By using the mean inter-item correlation coefficient and Cronbach's alpha coefficient, researchers can evaluate the scale reliability (35, 36). If the scale MIIC value is > 0.3, the internal reliability of the scale is acceptable (35). In this study, MIIC values of the entire BHWHPBAS-AV and all factors of BHWHPBAS-AV were all >0.3. A general criterion for a good internal reliability of scale is usually the Cronbach's alpha coefficient of ≥0.7 (26). In this research, Cronbach's alpha coefficients of the whole BHWHPBAS-AV and all factors of BHWHPBAS-AV were all > 0.7. Therefore, based on the above comprehensive analysis, BHWHPBAS-AV developed in this study has a satisfactory reliability.

## Limitations and Future Direction

Due to several limitations, future, studies should widen the scope of sampling. Moreover, BHWHPBAS-AV will be more widely verified and used in more areas of the country, so that BHWHPBAS-AV can be better revised and improved in the future. Owing to the lack of an appropriate golden criterion for measuring the adolescent haze weather health protection behavior, testing the criterion validity of BHWHPBAS-AV is a difficult task (29). Therefore, in the future, the criterion validity of BHWHPBAS-AV needs to be further evaluated. Furthermore, in the process of applying BHWHPBAS-AV in other countries, further cross-cultural BHWHPBAS-AV revision and cross-cultural BHWHPBAS-AV validation are also needed.

## Conclusion

In conclusion, our research team developed and validated the BHWHPBAS-AV with proven reliability and validity. BHWHPBAS-AV can be applied to evaluate adolescent health protection behaviors taken against haze weather. BHWHPBAS-AV can also help teachers at schools, as well as medical staff in community health care institutions, to carry out targeted behavioral interventions and to conduct health education for teenagers regarding protection against haze weather. Ultimately, this will help teenagers establish better haze weather health protection behaviors, which will allow for their healthy growth.

## Data Availability Statement

The datasets generated for this study are available on request to the corresponding author.

## Ethics Statement

The studies involving human participants were reviewed and approved by Health and Family Planning Commission of Hebei Province (NO. 20150072). The study was also approved by Medical Ethics Committee of Hebei University. Written informed consent to participate in this study was provided by the participants' legal guardian/next of kin.

## Author Contributions

QZ, CY, and LT conceived, designed the study, and wrote manuscript. CY, ST, YZ, HD, YC, and YL collected data, analyzed data, and revised manuscript critically. All authors contributed to the article and approved the submitted version.

## Conflict of Interest

The authors declare that the research was conducted in the absence of any commercial or financial relationships that could be construed as a potential conflict of interest.

## Abbreviations

BHWHPBAS-AV: Brief Haze Weather Health Protection Behavior Assessment Scale-Adolescent Version
CVI: Content validity index
MIIC: Mean inter-item correlation coefficient
EFA: Exploratory factor analysis
PCA: Principal component analysis
KMO: Kaiser-Meyer-Olkin
CFA: Confirmatory factor analysis
χ^2^: Chi square value
df: Degrees of freedom
χ^2^/df: Chi square value/degrees of freedom
RMSEA: Root-mean-square error of approximation
GFI: Goodness of fit index
NFI: Normed fit index
IFI: Incremental fit index
TLI: Tueker-Lewis index
CFI: Comparative fit index.
